# Six Short Welfare Conferences

**Published:** 1920-06-12

**Authors:** 


### Six Short Welfare Conferences.

A series of single-day conferences on maternity and child-welfare has been organised by the National Association for the Prevention of Infant Mortality and National Baby Week Council to take place in Leeds, Manchester, Bradford, Brighton, Crewe, and Wrexham. The Leeds conference is on June 30, and the others—with the exception of that at Crewe—are in the first week of July; Crewe is fixed for September 10. The subjects to be discussed are widows' pensions, the home and its substitutes, infant-welfare work in retrospect and forecast, and the "decay of parenthood." Among those who have promised to read papers are Mrs. H. B. Irving, Mrs. H. A. L. Fisher, Dr. C. W. Saleeby, and Dr. H. W. Pooler (medical officer for maternity and child-welfare to the County of Derby). Delegates are invited from any official or unofficial authorities who are interested, and the public may also attend.

